# The Human Nature of Music

**DOI:** 10.3389/fpsyg.2018.01680

**Published:** 2018-10-04

**Authors:** Stephen Malloch, Colwyn Trevarthen

**Affiliations:** ^1^Westmead Psychotherapy Program, Sydney Medical School, The University of Sydney, Sydney, NSW, Australia; ^2^The MARCS Institute for Brain, Behaviour, and Development, Western Sydney University, Sydney, NSW, Australia; ^3^Department of Psychology, School of Philosophy, Psychology and Language Sciences, The University of Edinburgh, Edinburgh, United Kingdom

**Keywords:** musicking, motor intelligence, gestural narrative, infant musicality, cultural learning

## Abstract

Music is at the centre of what it means to be human – it is the sounds of human bodies and minds moving in creative, story-making ways. We argue that music comes from the way in which knowing bodies (Merleau-Ponty) prospectively explore the environment using habitual ‘patterns of action,’ which we have identified as our innate ‘communicative musicality.’ To support our argument, we present short case studies of infant interactions using micro analyses of video and audio recordings to show the timings and shapes of intersubjective vocalizations and body movements of adult and child while they improvise shared narratives of meaning. Following a survey of the history of discoveries of infant abilities, we propose that the gestural narrative structures of voice and body seen as infants communicate with loving caregivers are the building blocks of what become particular cultural instances of the art of music, and of dance, theatre and other temporal arts. Children enter into a musical culture where their innate communicative musicality can be encouraged and strengthened through sensitive, respectful, playful, culturally informed teaching in companionship. The central importance of our abilities for music as part of what sustains our well-being is supported by evidence that communicative musicality strengthens emotions of social resilience to aid recovery from mental stress and illness. Drawing on the experience of the first author as a counsellor, we argue that the strength of one person’s communicative musicality can support the vitality of another’s through the application of skilful techniques that encourage an intimate, supportive, therapeutic, spirited companionship. Turning to brain science, we focus on hemispheric differences and the affective neuroscience of Jaak Panksepp. We emphasize that the psychobiological purpose of our innate musicality grows from the integrated rhythms of energy in the brain for prospective, sensation-seeking affective guidance of vitality of movement. We conclude with a Coda that recalls the philosophy of the Scottish Enlightenment, which built on the work of Heraclitus and Spinoza. This view places the shared experience of sensations of living – our communicative musicality – as inspiration for rules of logic formulated in symbols of language.

“There are certain aspects of the so-called ‘inner life’—physical or mental —which have formal properties similar to those of music—patterns of motion and rest, of tension and release, of agreement and disagreement, preparation, fulfilment, excitation, sudden change, etc. Langer (1942, p. 228).

“The function of music is to enhance in some way the quality of individual experience and human relationships; its structures are reflections of patterns of human relations, and the value of a piece of music as music is inseparable from its value as an expression of human experience” Blacking (1995, p.31).

“The act of musicking establishes in the place where it is happening a set of relationships, and it is in those relationships that the meaning of the act lies. They are to be found not only between those organized sounds which are conventionally thought of as being the stuff of musical meaning but also between the people who are taking part, in whatever capacity, in the performance” Small (1998, p.9).

## Prelude

We present a view that places our ability to create and appreciate music at the center of what it means to be human. We argue that music is the sounds of human bodies, voices and minds – our personalities – moving in creative, story-making ways. These stories, which we want to share and listen to, are born from awareness of a complex body evolved for moving with an imaginative, future seeking mind in collaboration with other human bodies and minds. Musical stories do not need words for the creation of rich and inspiring narratives of meaning.

We adopt the word ‘musicking’ (as used above by Christopher Small) to draw attention to the embodied energy that creates music, and which moves us, emotionally and bodily. Further, we argue that music comes from the way in which *knowing bodies* (Merleau-Ponty, 2012 [1945], p. 431) prospectively explore the environment using habitual ‘patterns of action,’ which we have identified as our innate ‘communicative musicality,’ observed while infants are in intimate communication with loving caregivers (Malloch and Trevarthen, 2009a). In short case studies of infant interactions with micro analyses of video and audio recordings, we show communicative musicality in the timings and shapes of intersubjective vocalizations and body movements of adult and child that improvise with delight shared narratives of meaning.

Following a survey of the history of discoveries of infant abilities, we propose that the gestural narrative structures of voice and body seen as infants communicate with loving caregivers, ‘protonarrative envelopes’ of expression for ideas of activity (Panksepp and Bernatzky, 2002; Panksepp and Trevarthen, 2009), are the building blocks of what become particular cultural instances of the art of music, and of dance, theater and other temporal arts (Blacking, 1995).

As the child grows and becomes a toddler, she or he eagerly takes part in a children’s musical culture of the playground (Bjørkvold, 1992). Soon more formal education with a teacher leads the way to the learning of traditional musical techniques. It is at this point that the child’s innate body vitality of communicative musicality can be encouraged and strengthened through sensitive, respectful, playful, culturally informed teaching (Ingold, 2018). On the other hand, it may wither under the weight of enforced discipline for the sake of conforming to pre-existing cultural rules without attention to the initiative and pleasure of the learner’s own music-making.

The central importance of our abilities for music as part of what sustains our well-being is supported by evidence that communicative musicality strengthens emotions of social resilience to recover from mental stress and illness (Pavlicevic, 1997, 1999, 2000). Drawing on the experience of the first author as a counselor, we argue that the strength of one person’s communicative musicality can support the vitality of another’s through the application of skilful techniques that encourage an intimate, supportive, therapeutic, spirited companionship.

Turning to brain science, focussing on hemispheric differences in performance and in response to music, and the affective neuroscience of Jaak Panksepp (1998), we emphasize that the psychobiological purpose of our ‘muse within’ (Bjørkvold, 1992) grows from the integrated rhythms of neural energy for prospective, sensation-seeking affective guidance of vitality of movement in the brain (Goodrich, 2010; Stern, 2010).

We conclude with a Coda – an enquiry into the philosophy of the Scottish Enlightenment, which built on the work of philosophers Heraclitus and Spinoza. This view of living in community gives innate sympathy or ‘feeling with’ other humans a fundamental role within a duplex mind seeking harmony in relationships by attunement of motives (Hutcheson, 1729, 1755). It places the shared experience of sensations of living – our communicative musicality – ahead of logic formulated in symbols of language.

## Music Moves US – Embodied Narratives of Movement

Small (1998) calls attention to music as intention in activity by using the verb *musicking –* participating as performer or listener with attention to the sounds created and the appreciation and participation by others. The compelling quality of music comes from the relationships of sounds, bodies and psyches. ‘Musicking’ points to our musical life in active ‘I-Thou’ relationships. Only in this intimacy of consciousness and its interests can we share ‘I-It’ identification and use of objects, giving things we use, including musical compositions, meaning (Buber, 1923/1970).

‘I-Thou’ relationships are entered into through the body. The philosopher Merleau-Ponty (2012 [1945]) writes that “The subject only achieves his ipseity [individual personality, selfhood] by actually being a body, and by entering into the world through his body… The ontological world and body that we uncover at the core of the subject are not the world and the body as *ideas*; rather, they are the world itself condensed into a comprehensive whole and the body itself as a *knowing-body*” (p. 431; italics added). Musicking is *knowing bodies* coming alive in the sounds they make. Scores and other tools that record the product of musicking, performed or imagined, aid the retention of ideas, as semantics of language does, and they serve discussion and analysis – but they are not the same as the breathing, moving, *embodied* experience of human musicking (Mithen, 2005).

Musicking is the expression of the sensations of what we call our *communicative musicality*, for the purpose of creating music that is enlivening and ‘beautiful’ (Malloch, 1999; Malloch and Trevarthen, 2009a; Trevarthen, 2015; Trevarthen and Malloch, 2017b). We define communicative musicality as our innate skill for moving, remembering and planning projects in sympathy with others through time, creating an endless variety of dramatic temporal narratives in song or instrumental music. We describe this life-sharing in movement as having three components:

*Pulse* – a regular succession through time of discrete movements (which may, for example, be used to create sound for music, or to create movement with music – dance) using our felt sense of acting which enables the ‘future-creating’ predictive process by which a person may anticipate or create what happens next and when.

*Quality* – consisting of the contours of expressive vocal and body gesture, shaping our felt sense of time in movement. These contours can consist of psychoacoustic attributes of vocalizations – timbre, pitch, volume – or attributes of direction and intensity of the moving body perceived in any modality.

*Narratives* of individual experience and of companionship, built from sequences of co-created gestures which have particular attributes of *pulse* and *quality* that bring aesthetic pleasure (Malloch and Trevarthen, 2009b; Trevarthen and Malloch, 2017b).

With music we create memorable poetic events in signs that express in sound our experience of living together in the creating vitality of ‘the present moment’ (Stern, 2004, 2010). The anthropologist and ethnologist Claude Lévi-Strauss draws attention of linguists to the structured ‘raw’ emotive power of music (beyond what words may be ‘cooked’ to say).

“In the first volume of the *Mythologiques, Le Cru et le Cuit.* Lévi-Strauss refers to music as a unique system of signs possessing ‘its own peculiar vehicle which does not admit of any general, extramusical use’. Yet he also allows that music has levels of structure analogous to the phonemes and sentences of language. The absence of words as the connecting level is an obvious and pertinent fact in the structuring of meaning within music as a sign system.” (Champagne, 1990, p. 76).

Goodrich (2010), in her appreciation of the contribution of neuroscientists Llinás (2001) and Buzsáki (2006) to the science of the mind for skilled movement, cites Llinás’ evidence on the role of intuitive structural ‘rules,’ seen also in a musical performance.

“Llinás describes another method of keeping movement as efficient as possible: motor ‘Fixed Action Patterns’ (FAPs), distinct and complicated ‘habits’ of movement built from reflexes, habits that we develop to streamline both neural action and muscle movement. These are not entirely fixed, despite their name; they are constantly undergoing modification, adaptation, refinement, and they overlap each other… Llinás even argues that the extraordinarily precise motor control of Jascha Heifetz playing Tchaikovsky’s violin concerto is composed of highly elaborated and refined FAPs, a description most instrumentalists would find absolutely plausible” (Goodrich, 2010, p. 339).

As Llinás himself writes,

“Can playing a violin concerto be a FAP? Well, not all of it, but a large portion. Indeed, the unique and at once recognisable style of play Mr. Heifetz brings to the instrument is a FAP, enriched and modulated by the specifics of the concert, generated by the voluntary motor system” (Llinás, p. 136).

We add that skilled FAPs are not “composed of reflexes” as separate automatic responses. Rather they are purposeful projects that are animated to be developed imaginatively, and affectively, with exploration of their biomechanical “degrees of freedom,” as in Nikolai Bernstein’s detailed description of how a toddler learns to become a virtuoso in bipedal locomotion, which he calls *The Genesis of the Biodynamical Structure of the Locomotor Act* (Bernstein, 1967, p. 78). The testing of these locomotor acts is with an immediate and essential estimation by *gut feelings* (Porges, 2011) of any risks or benefits, any fears or joy, they may entail within the body.

## Music Reflects the Felt-Sense of our Future-Exploring Motor Intelligence

Consciousness is created as the ongoing sense of self-in-movement with which we experience and manipulate the world around us. Its origin is in our evolutionary animal past, evolved for new collaborative, creative projects, regulated between us by affective expressions of feelings of vitality from within our bodies (Sherrington, 1955; Panksepp, 1998; Damasio, 2003; Mithen, 2005; Stern, 2010; Eisenberg and Sulik, 2012).

Using the philosopher and psychologist James (1892/1985) as a starting point for exploring the intimacy of feeling that supports and guides psychotherapy, Russell Meares (2005, p.18), following the ‘conversational model’ of therapy developed with his collaborator psychiatrist/psychotherapist Hobson (1985), identifies five dimensions of the self:

1. *awareness* which is necessary for the experience;
2. there is a *shape* to this inner life;
3. there is *a sense of its ongoingness*;
4. our inner life has a *connectedness or unity*;
5. our experience of our inner life goes on inside a virtual container which is *our background emotional state* and the *background experience of our body*.

These sensuous qualities of the experienced self are expressed in music, and in other temporal arts, as ‘the human seriousness of play’ (Turner, 1982). Music, as Susan Langer says so clearly in the quote at the start of this paper, has qualities of this inner life described by Meares as shape, ongoingness and flow, connectedness and unity. The notion of music as expressive of the movements of our inner life has also been explored by music theorists, most notably Ernst Kurth (1991). Likewise, in his book *Self comes to Mind*, Antonio Damasio likens all our emotion and feeling to a ‘musical score’ that accompanies other ongoing mental process (Damasio, 2010, p.254).

The ultimate motivation for creating music can even be traced to the cellular level. In *Man On His Nature* (Sherrington, 1955), in a chapter entitled *The Wisdom of the Body*, the creator of modern neurophysiology Charles Sherrington called the coming together of communities of cells into the integrated body, nervous system and brain of a person “an act of imagination” (p. 103). Neuroscientist Rudolfo Llinás also grants subjectivity, a sense of self, to all forms of life. “Irritability [i.e., responding to external stimuli with organized, goal-directed behavior] and subjectivity, in a very primitive sense, are properties originally belonging to single cells” (Llinás, 2001, p.113). “Thinking”, writes Llinás (2001, p.62), “ultimately represents movement, not just of body parts or objects in the external world, but of perceptions and complex ideas as well.”

Intrinsic to the sociability of this intelligence of movement is sensitivity for the exploration of the future, which is woven into our creation and experience of music. Karl Lashley (1951) reflecting on the evolution of animal movement, proposed that the ability to predict what might come next, and to plan the ‘serial ordering’ of separate actions, may be understood as the foundation for our logical reasoning as an individual, as it is for the grammar or syntax and prosody of our communication in language. It is essential for musicking. A restless future-seeking intelligence, with our urge to share it, inspires us to express our personalities as ‘story-telling creatures,’ who want to share, and evaluate, others’ stories (Bruner, 1996, 2003).

All animal life depends on motivated movement – the urge to explore with curiosity – to move towards food with anticipation, to move away from a predator with fear, to interact playfully with a trusted friend (Eibl-Eibesfeldt, 1989; Panksepp and Biven, 2012; Bateson and Martin, 2013). A great achievement of modern science of the mind was the discovery by a young Russian psychologist Nikolai Bernstein of how all consciously made body movements depend upon an ‘image of the future’ (Bernstein, 1966, 1967).

Bernstein applied the new technology of movie photography to make refined ‘cyclographic’ diagrams of displacements of body parts, from which he analyzed the forces involved to fractions of a second. His findings reported in *Coordination and Regulation of Movements* became widely known in English translation in 1967, at the same time as video records of infant behaviors were described more accurately (see next Section The Genesis of Music in Infancy – A Short History of Discoveries), revealing their anticipatory motor control adapted for intelligent understanding of how objects may be manipulated, as well as for communication and cooperation (Trevarthen, 1984b, 1990b).

Our musical creativity and pleasure come from the way our body hopes to move, with rhythms and feelings of grace and biological ‘knowing’ (see Merleau-Ponty). The predicting, embodied self of a human being experiences time, force, space, movement, and intention/directionality in being. Together, these form the Gestalt of ‘vitality’ (Stern, 2010), the ‘forms of feeling’ (Hobson, 1985) by which we sense in ourselves and in others that movement, be that movement of the body or of a piece of music, is ‘well-done’ (Trevarthen and Malloch, 2017b).

## The Genesis of Music in Infancy – A Short History of Discoveries

The ability to create meaning with others through wordless structured gestural narratives, that is, our communicative musicality, emerges from before birth and in infancy. From this innate musicality come the various cultural forms of music.

Any attempt to understand how human life has evolved its unique cultural habits needs to start with observing what infants know and can do. Organisms regulate the development of their lives by growing structures and processes from within their vitality, by *autopoiesis* that requires anticipation of adaptive functions. And they must develop and protect their abilities in response to environmental affordances and dangers, with *consensuality* (Maturana and Varela, 1980; Maturana et al., 1995). Infants are ready for human cultural invention and collaboration as newly hatched birds are ready for flying – within ‘the biology of love’ (Maturana and Varela, 1980; Maturana and Verden-Zoller, 2008). All organisms reach out in time and space to make use of the ‘affordances’ for thought and action (Gibson, 1979).

**FIGURE 1:**
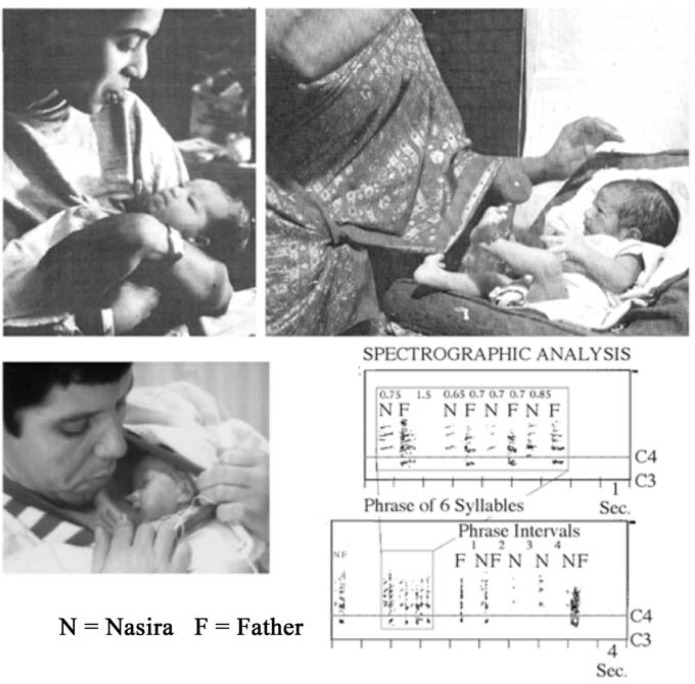
Inborn musicality shared in movement. Upper Left: Infant less than one hour after birth watches her mother’s tongue protrusion and imitates. Upper Right: On the day of birth, a baby in a hospital in India shares a game with a woman who moves a red ball. The baby tracks it with coordinated movements of her eyes and head, both hands and one foot. Lower: A two-month premature girl, Nasira, with her father, who holds her against his body in ‘kangaroo’ care. They exchange short ‘coo’ sounds with precisely shared rhythm. Upper Left and Upper Right: Photos for own use of second author from colleagues Vasudevi Reddy and Kevan Bundell. Part reproduced from Trevarthen (2015), Figure 1, p. 131. Lower: Photo from Trevarthen (2008), Figure 2, p. 22. Original spectrograph in Malloch (1999), Figure 3, p. 37.

Infants have no language to learn what other humans know, or what ancestors knew. But the vitality of their spontaneous communicative musicality, highly coordinated and adapted to be shared through narratives with sympathetic and playful companions, enables meaningful communication in the ‘present moment’ (Stern, 2004; **Figure 1**, Upper Right), which may build serviceable memories extended in space and time (Donaldson, 1992).

In this section we review changes in understanding of infant abilities over the past century which can help explain the peculiar way music has in the past been seen by some leading psychologists and linguists as a relatively insignificant epiphenomenon, learned for play, not, as we argue in this paper, a source of all talents for communication, rooted in our innate human communicative musicality of knowing bodies (Merleau-Ponty, 2012 [1945]) moving with prospective intuition to engage the world in company, ‘intersubjectively,’ from the start (Zeedyk, 2006).

Two leading scholars in medical science and the science of child development in the past century, Freud (1923) and Piaget (1958, 1966), declared that infants must be born without conscious selves conceiving an external world, and unable to adapt their movements to the expressive behavior of other people. The playful and emotionally charged behaviors of mothers and other affectionate carers were considered inessential to the young infant, who needed only responses to reflex demands for food, comfort and sleep.

Then René Spitz (1945) and Bowlby (1958) revealed the devastating effects on a child’s emotional well-being of separation from maternal care in routine hospital care with nursing directed only to respond to those reflex demands. Spitz observed that babies develop smiling between 2 and 5 months to regulate social contacts (Spitz and Wolf, 1946), and he went on to study the independent will of the baby to regulate engagements of care or communication, by nodding the head for ‘yes’ or shaking for ‘no’ (Spitz, 1957).

In the 1960s a major shift in understanding of the creative mental abilities of infants was inspired by a project of the educational psychologist Bruner (1966, 1968), and the pediatrician Brazelton (1961, 1979), who perceived that infants are gifted with sensibilities for imaginative play and ready to start cultural learning from the first weeks after birth. Supported by insights of Charles Darwin and by new findings of anthropology and animal ethology they studied infant initiatives to perceive and use objects, and they were impressed by the intimate reciprocal imitation that develops between infants and affectionate parents and caregivers who offer playful collaboration with the child’s rhythms and qualities of movement. Film studies showed that young infants make complex shifts of posture and hand gestures that are regulated rhythmically, similar to the same movements of adults (Bruner, 1968; Trevarthen, 1974).

While this new appreciation of infant abilities was developed at the Center for Cognitive Studies at Harvard, radically transforming the ‘cognitive revolution’ that was announced there by George Miller, Noam Chomsky and Jerome Bruner in 1960, nearby at the Massachusetts Institute of Technology, a project initiated by Bullowa (1979) sought evidence on the behaviors that regulate dialog before language. Bullowa used information from anthropology to draw attention to the *measured dynamics* of communication.

“For an infant to enter into the sharing of meaning he has to be in communication, which may be another way of saying *sharing rhythm*…. The problem is how two or more organisms can share innate biological rhythms in such a way as to achieve communication which can permit transmission of information they do not already share.” (Bullowa, 1979, p. 15, italics added).

Wanting to understand how the rhythmic flow of dialog can be shared with a child too young for speech, she pointed a way to the appreciation of the role of human communicative musicality as inspiration for the development of behaviors for carrying meaning in language – thinking and communicating in words that will be acquired to specify facts, and to describe and think about how these facts are related or may be used.

The importance of rhythm and the graceful narratives of movement displayed by infants as they communicate purposes and feelings was revealed sixty years ago by a psychobiological approach using photography and movie film, then video. Discoveries were made that challenged the theory that infants had no minds, no sense of self, and therefore no sense of others (Zeedyk, 2006; Reddy, 2008). Most astonishing, and dismissed with derision by convinced rational mind-separate-from-body constructivists, was the finding that infants activate the many parts of their body with an exquisite sense of time, and that they can use the rhythms of expression skilfully to imitate in inter-synchrony with attentive responses from an adult (**Figure 1**).

In his work as a pediatrician, Brazelton (1961), developing his now famous Neonatal Assessment Scale (Brazelton, 1973; Brazelton and Nugent, 1995), accepted and encouraged the natural love mother and father felt for their new baby, and showed how appreciative the baby could be of their actions to each other and to the baby. This welcoming of the newborn as a person with intelligence and sociable impulses confirmed the parents’ belief that they could communicate feelings and interests by responding to their baby’s exquisitely timed looks, smiles, hand gestures and cooing with their own exquisitely timed gestures of voice and body. It transformed medical concern for the baby. As Brazelton declared in Margaret Bullowa’s book, “The old model of thinking of the newborn infant as helpless and ready to be shaped by his environment prevented us from seeing his power as a communicant in the early mother-father-infant interaction. To see the neonate as chaotic or insensitive provided us with the capacity to see ourselves as acting ‘on’ rather than ‘with’ him” (Brazelton, 1979, p.79).

New attention to newborns within hours of their delivery, with the aid of films, led to confirmation that the baby could imitate adult expressions with careful timing of movements of eyes, face, mouth and hands (Maratos, 1973, 1982; Meltzoff and Moore, 1977; Kugiumutzakis, 1993; Nagy and Molnár, 1994, 2004; Nagy, 2011; Kugiumutzakis and Trevarthen, 2015; **Figure 1**, Upper Left). The findings proved that the baby is born with an *altero-ceptive awareness* of another person’s body parts as having feelings in movement like their own *proprio-ceptive* ones. It also became clear that this consciousness appreciates the balance and drama of a collaborative narrative flow with shared rhythms – essential to our ability for musicking (Trevarthen, 1974, 1977, 2005a).

The pediatrician Sander, (1964, 1975; republished in Sander, 2008) recognized that an infant and caregiver create a coherent system of actions regulated with feelings of vitality in shared time. This dynamic collaboration was also discovered by Daniel Stern when he examined recordings of a mother playing with her three-month-old twins (Stern, 1971).

A stimulating contribution to this new approach came from the work of anthropologist and linguist Mary Catherine Bateson, daughter of anthropologists Gregory Bateson and Margaret Meade. In 1969 Bateson had her first child after beginning postgraduate studies at MIT with Margaret Bullowa, researching language development using statistical analysis of vocal expressions. Observing a film in Bullowa’s collection as well as the experience of rich exchanges with her own infant opened her awareness of the form and timing of communication that developed in the first 3 months, which she called ‘proto-conversation’ (Bateson, 1971). She benefitted from attention to the field studies of Albert Scheflen on the stream of conversation (Scheflen, 1972) and Ray Birdwhistell on body movements in natural conversation (Birdwhistell, 1970).

Reviewing her work in Bullowa’s book, she said:

“… the mother and infant were collaborating in a pattern of more or less alternating, non-overlapping vocalization, the mother speaking brief sentences and the infant responding with coos and murmurs, together producing a brief joint performance similar to conversation, which I called ‘proto conversation’. The study of timing and sequencing showed that certainly the mother and probably the infant, in addition to conforming in general to a regular pattern, were acting to sustain it or to restore it when it faltered, waiting for the expected vocalization from the other and then after a pause resuming vocalization, as if to elicit a response that had not been forthcoming. These interactions were characterized by a sort of delighted, ritualized courtesy and more or less sustained attention and mutual gaze. Many of the vocalizations were of types not described in the acoustic literature on infancy, since they were very brief and faint, and yet were crucial parts of the jointly sustained performances.” (Bateson, 1979, p. 65).

Bateson’s work confirmed Bruner’s realization that Noam Chomsky’s hypothesis of a specific Language Acquisition Device (LAD) or innate ability of the child to construct syntax, the grammatical order of words, to formulate ideas (Chomsky, 1965), paid no heed to the vital importance of a complementary ability of a parent to encourage enrichment of reference in communication with a young child, a Language Acquisition Support System (LASS). As Bruner, expressing his psychology of education, put it “the LADD needs a LASS” (Bruner, 1983). Child and adult share rules of imagination for all kinds of movement, including spoken propositions.

A follower of Chomsky’s theory of the evolution of language as reasoning, Stephen Pinker, in his perhaps overly ambitiously titled *How the Mind Works* (Pinker, 1997) claimed, “As far as biological cause and effect are concerned, music is useless.” He gave no attention to movement and time, the communication of infants, playful children, affectionate parents, the poetry of music, or Einstein’s theory of his mathematical invention as “sensations of bodily movement” (Hadamard, 1945), thus misunderstanding the origins and purpose of rational discourse (see Sections Communicative Musicality and Resilience of the Human Spirit and Musical Affections of the Embodied Human Brain).

We now have evidence from many studies analyzing behaviors that demonstrate that infants show a rich spectrum of expressive movements of the upper parts of their bodies (Trevarthen, 1984a), not just the ‘categorical emotions’ identified by Paul Ekman (Ekman and Friesen, 1975), but the ‘complex social emotions’ that Damasio (1999, 2010) describes as regulators of well-being in intimate interpersonal relations, and expression of a moral personality in society – expressions of such feelings as embarrassment, shame, guilt, contempt, compassion, and admiration.

Most importantly, study of recordings reveal that modulations of timing, of rhythms, and of the flow of vitality forms shared with infants have the characteristics recognized as musical (Trevarthen, 1990a). These have been precisely defined by acoustic analysis of vocalizations of adult and infant in dialogs and games (Malloch et al., 1997; Malloch, 1999; Trevarthen, 1999; Trehub, 2003).

## Case Studies of Infant Musicality

We summarize here key findings related to the growth of musical abilities from studies of infant individuals that we have reported previously.

First there is evidence from a recording made by Saskia van Rees in an intensive care unit in Amsterdam (van Rees and de Leeuw, 1993) that rhythms corresponding to those of human locomotion are present in vocalizations of a premature infant which are precisely coordinated with simple vocal exchanges with a caring father (**Figure 1**, Lower).

The recording of a two-month premature girl with her father, who was holding her under his clothes against his body in ‘kangaroo’ care, shows that they exchange short ‘coo’ sounds, the father imitating her sounds, with precise timing based on a comfortable walking rhythm of *andante* – one step every 0.7 s. Father (F) and the baby Naseera (N) are equally precise in their timing, which also shows what a phonetician would recognize as a ‘final lengthening’ characteristic of a spoken phrase - when they are ready to stop the dialog the interval lengthens to 0.85 s. Following the shared phrase with its syllable-length durations, they exchange single sounds separated at 4 s intervals, the normal duration of a short spoken phrase. The recording supports our contention that even a prematurely born baby is skilled in sharing a musical pulse (Trevarthen, 2009).

**FIGURE 2:**
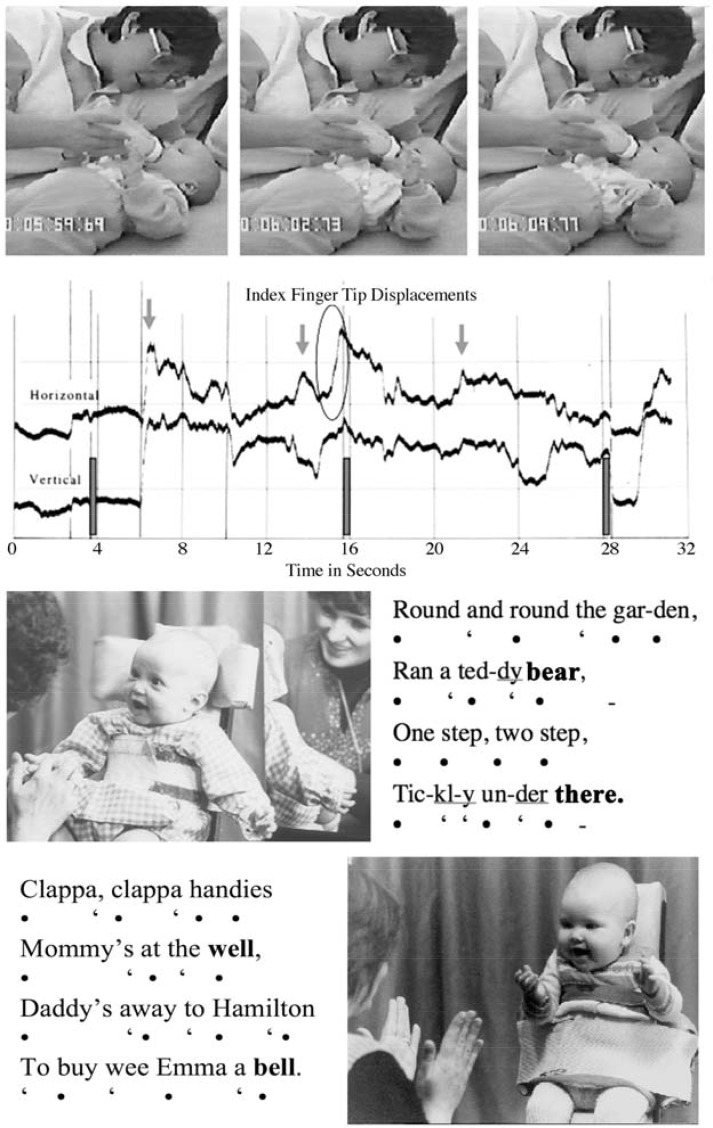
Communicating in the rhythm of narratives with baby songs. Upper: A five-month-old girl, who is totally blind, ‘conducts’ her mother’s singing with her left hand. The graph of her hand movements with her mother’s voice marks, with black bars, three moments in the narrative, at 4, 16, and 28 s, where the infant anticipates the mother’s voice by 0.3 s. These are times, 12 s apart, when lines of the slow lullaby commence. The arrows identify moments when there is perfect synchrony between the mother’s voice and the baby’s index finger. The circled index finger movement is 300 msec ahead of a lift in the pitch of the mother’s voice. Lower: Two infants share the poetry of action songs with their mothers. A four-month-old enjoys her mother’s singing of a song and accompanies the narrative with movements of her baby’s hand. And a six-month-old imitates clapping movements in synchrony with the song. Both songs show an iambic rhythm of short and long syllables, and rhyming vowels, ‘bear’ with ‘there’ and ‘well’ with ‘bell,’ which the babies imitate with their voices. (Upper: Analyses of video from Professor Gunilla Preisler, University of Stockholm. Previously published in Trevarthen (1999) and Schögler and Trevarthen (2007). Lower: From Trevarthen (2015), modified from Figure 4, p.137.)

A recording with a blind 5-month-old girl illustrates intermodal attunement between the heard melody of a mother’s song and the proprioceptive feelings in the body of the baby of a gesturing left arm and hand (**Figure 2**, Upper). The human ability to sense the shape of a melody within the body is intrinsic to our enjoyment of music as human communication (Stern, 2010). Maria is totally blind and has never seen her hand. Maria and her mother were assisting in a project of Professor Gunilla Preisler in Stockholm to aid communication with blind and deaf infants.

While her baby is lying down during bottle feeding, the mother sings two baby songs including “Mors Lille Olle,” well-known throughout Scandinavia. It was not realized until later when the video was viewed that Maria was ‘conducting’ the melodies with delicate expressive movements of her left hand, while the right hand was making unrelated movements, stroking her body. When Professor of Music at Edinburgh University, Nigel Osborne, saw the film he said, “Yes she is conducting using the conventional movements of a professional conductor, describing a phrase with a sweeping movement, pointing up for a higher pitch, and dropping her wrist at the close of a verse – and she is making the movements with some anticipation.” Microanalysis supported what he observed. At certain points in the course of the melody Maria’s finger moves 300 milliseconds before the mother’s voice. She knows the song well, and leads the ‘performance’ (Trevarthen, 1999; Schögler and Trevarthen, 2007).

Although blind, Maria knows the feelings of anticipated movement of her hand, and uses them to sense and share the human vitality dynamics in her mother’s voice. This *kinematic* sensibility was identified by Olga Maratos in her pioneering research in imitation as foundational for the ability of a young infant to reproduce another person’s expression seen or heard (Maratos, 1973, 1982). Indeed, vocal perception, detecting the modulation of pitch and timing in an adult’s voice sounds, develops much faster than vocal production. The infant may be tracking sound with reference to the kinesics of the fastest and most complex gestural movements of her hands.

**FIGURE 3:**
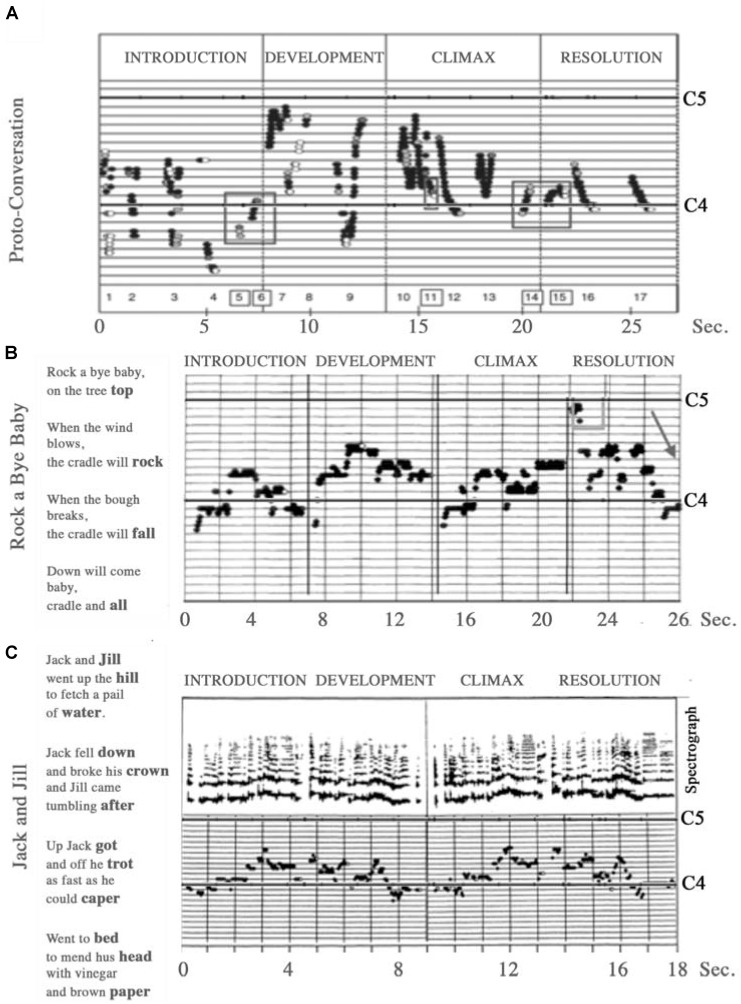
Stories of changing pitch in the singing voice. These examples illustrate the narrative phases that infants respond to, which are found in infant directed speech or baby songs in different languages. **(A)** A proto-conversation of a mother with a 6-week-old. The baby shares the development, climax and resolution of the narrative, with vocalizations close to middle C. **(B)** A slow lullaby, of 26 s, has four-line stanzas, with rhyming vowels. **(C)** A more animated song of 18 s shows the same poetic organization with undulations of pitch and vowels rhyming between first and second lines and between final words of each verse. **(A)** Original in Malloch (1999), Figure 5, p. 41. **(B)** From Trevarthen (2015), a portion of Figure 4, p. 137, and Trevarthen (2016), Figure 3, p. 10. Original in Malloch (1999), Figure 10, p. 49. **(C)** Original in Trevarthen (1999), Figure 2, p. 183.

When taking part in a nursery song, infants demonstrate sensitivity for melodic phrase structure, attending to the rhyming vowels at the ends of lines, and by 5 months the infant can vocalize a matching vowel in synchrony with the mother (Trevarthen, 2008). For our final example, the application of acoustic analysis with observations on gestural behaviors of infants in the middle of the first year, suggests that melodic patterns, common to different cultures, define four-line verses (Imberty, 2000), with a pattern of Introduction, Development, Climax and Resolution, identified in proto-conversations with two-month-olds (**Figure 2**, Lower, and **Figure 3**), and we note similarities with the sections recognized in classical Roman rhetoric or speech-making – *exordium, narratio, confutatio*, and *confirmatio*. In spite of very different conventions in musical performances in different communities, a parent, or a child, wanting to share the pleasure of songs and action games with a baby, naturally adopts the intuitive formula of a poetic verse to share a story of body movement.

**FIGURE 4:**
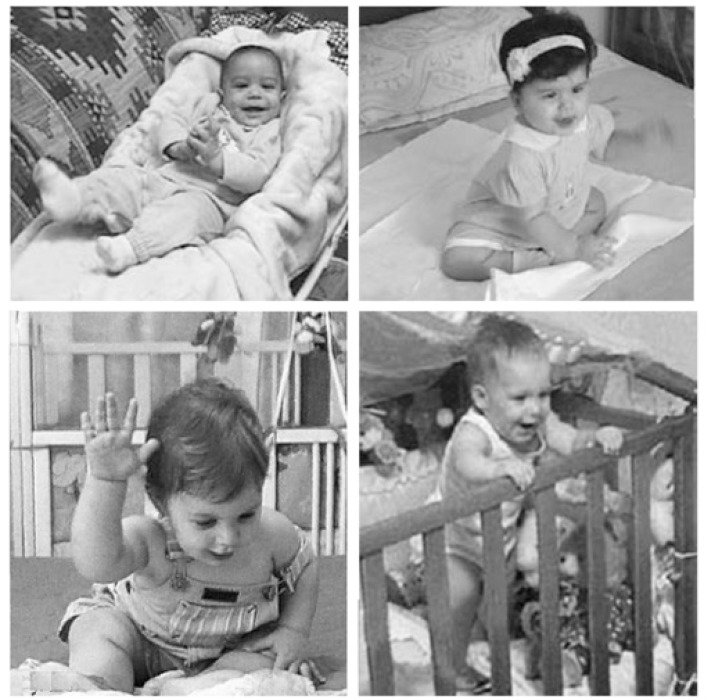
Katerina Mazokopaki’s baby dancers: Upper Left: Georgos, 3.5 months lying on a comfortable bed, responds with a big smile and gestures of hands and feet to accompany the music. Upper Right: Katerina, 9 months, smiles, bounces and extends her arms to ‘fly’ into action. Lower Left: Panos, 9 months, smiles his greeting, then beats the floor rhythmically with his right hand. Lower Right: Anna, 10 months, standing in her cot, smiles and starts dancing vigorously, swinging her bottom. All four sing with the music (Photos supplied to the CT).

Lastly, we present the work of Katerina Mazokopaki, a developmental psychologist who is a pianist and teacher of piano playing. She made a study of babies in Crete with her professor, Giannis Kugiumutzakis, an expert in analysis of imitative games with newborns (Mazokopaki and Kugiumutzakis, 2009). The babies were left alone in a familiar place at home amusing themselves. Then a recording of a Greek baby song came on. Between 3 and 10 months old they all reacted in the same way. First they looked surprised; then they looked about as if someone had come into the room; and finally they smiled with delight and started performing with the music, inspired by the pulse and melody, joining the music with their different abilities to dance and sing (**Figure 4**).

## Communicative Musicality and Education into the Culture of Music

Mastery of a musical culture, and of language, starts with the intuitive vocal interactions between caregiver and infant (Vygotsky, 1966). Our innate communicative musicality is the ‘raw material’ for cultural forms of music and the rules of grammar and syntax. A child makes stories in sound as an active participant whose pride to belong to the rich musical traditions of society propels them into learning and creating. This is the cultivation of communicative musicality to music, from innate self-expression to cultural practice and a musical identity (MacDonald et al., 2002; MacDonald and Miell, 2004). It is brought to life, as language is, with the enthusiastic support of more experienced companions (Bruner, 1983).

The desire for cultural participation is evident in informal learning in which children’s own musical culture grows from the vitality of *The Muse Within* (Bjørkvold, 1992). It is nurtured from the music, live and recorded, that the child hears all around and contributes to spontaneously, along with the invention of talking and verse-making with playmates, often accompanied by rhythmic stamping, hopping and jumping (Chukovsky, 1963). Our earliest shared signing of communicative musicality in infancy becomes dialogic ‘musical babbling’ from around 2 months old (Trevarthen, 1990a). Already at 2 months the infant is learning the cultural gestures and preferences that become the tools through which cultural meaning will be created and exchanged (Moog, 1976; Tafuri and Hawkins, 2008). Lullabies are sung more often in cultures that value quiet infants, playsongs in cultures that value lively enthusiasm in infants (Trehub and Trainor, 1998). A musical ‘proto-habitus’ is created (Gratier and Trevarthen, 2008).

The infant is born ready to interact and discover her musical culture. Hearing responds to musical sounds from the third trimester of pregnancy (Busnel et al., 1992), and infants can recognize music they heard before birth (Hepper, 1991). They recognize musical contours and rhythmic patterns (Trehub and Hannon, 2006), and ‘dance’ to music before they are one year old (Zentner et al., 2010). Infant-inclusive singing is preferred, like infant-inclusive speaking^1^.

From about 3 months of age in many (probably all) cultures, mothers start to sing baby songs (Trevarthen, 1986) to their infants. In a previous publication the second author summarized their characteristics:

(1) “The beat is clear and precisely modulated; usually in the *andante range* near *moderato (90-100* beats per minute).
(2) Often the beat is marked by clapping, or by regular movements of the baby’s body.
(3) The mother sings with a clear melodic tone varying her pitch in a treble range and making rises and falls to make a simple development of the ‘emotional narrative’.
(4) Songs commonly have a clear stanza form — usually 4 lines, each of 3 or 4 beats, making a verse (which therefore lasts 8-15 seconds).
(5) Especially in the last line, well controlled musical tricks varying the beat (*rubato*, *sforzando, ritardando, accelerando*) are used to mark a climax and resolution.” (Trevarthen, 1989, p. 96).

More recognizable musical forms grow with the spontaneous singing of young children as they play alone or with others (Barrett, 2011), practicing their musical craft. The Norwegian musicologist Jon-Roar Bjørkvold (1992) collected and studied the songs of 4–7-year-olds in three kindergartens in Oslo. He observed how they gave voice to emotion, conveyed information, and established relationships through learning and creating their own children’s musical culture. He identified two types of children’s singing – ‘egocentric’ for private pleasure, which, as the child matures, gives way to more social or ‘communicative’ music making.

As young children mature so they use their voice with a singing kind of expression in progressively more ‘symbolic’ ways. *Fluid/Amorphous Songs* “evolve in a completely natural way from the infant’s babbling as part of its first playful experiments with voice and sound. This type of spontaneous song, with its fanciful glissandi, micro-intervals, and free rhythms, is quite different from what we adults traditionally identify as song.” (Bjørkvold, 1992, p.65). *Song Formulas*, such as teasing songs, are symbolic forms for communicating and they flourish after the child begins to play with peers, typically at two or three. Elements of musically more complex *Standard Songs* are picked up from play with adults and hearing them sing, and are adapted to fit what the child is doing. This progressive ‘ritualisation’ of vocal creativity clarifies the adaptive motives for learning to sing, and how they express increasing narrative imagination for sharing ideas in culturally specific ways (Gratier and Trevarthen, 2008; Eckerdal and Merker, 2009), paralleling the way language is mastered (Chukovsky, 1963).

All through the development of children’s singing, repetition and variation, basic tools of any piece of music (for example, see Ockelford, 2017), are primary features as children explore the possibilities of musical form. Repetition and variation between the vocalizations of infant and caregiver feature from the very first shared vocalizations, regulating feelings in social interactions (Malloch, 1999). Later, the growing child will continue to play with how music can convey affect and change their own and others’ mood, the four-part structure of Introduction, Development, Climax and Resolution, identified above in the structure of a proto-conversation, becoming the basis of large scale musical works, as well as verbal argument (for example, see the sections of classical rhetoric).

How the child’s spontaneous musicality, as it grows in group practice without formal training (MacDonald and Miell, 2004), is received by the surrounding educational culture, is a vital ingredient in the child’s emerging ‘musical identity.’ Musical identity and self-efficacy or mastery of skill in music making inform each other, in reciprocal relationship. A child who sees themselves as a competent musician may attempt to learn a difficult piece of music, and their success at performing this piece will further bolster their sense of competence. And the way a child is welcomed into their musical culture is of vital importance as to whether this child thrives playfully with the musical tools at her disposal, developing her skill in the use of these tools, or shrinks away in disinterest because her own intrinsic musicality is not being heard or valued. If education does its job well, with the child as collaborative artist and thinker (Trevarthen et al., 2018), our rich inner narrative of affective life, generated with our prospective awareness of body movement, is expressed in our social group to create a life-affirming, inclusive culture of shared artful rituals that celebrate the aesthetic grace and moral graciousness of joy in performance (Frank and Trevarthen, 2012; Trevarthen and Bjørkvold, 2016). For example, the InCanto project (Tafuri and Hawkins, 2008) is a wonderful example of infants’ and parents’ being encouraged to have their expression of music cultivated in such a way that the infant grows into a child who shows greater ability to sing in tune, a greater range of musical expression, and overall more enthusiasm for music participation.

Problems from introducing an emphasis on enforced cultural learning too early are demonstrated by Bjørkvold (1992) who studied the musical games of children in Oslo, Moscow, St Petersburg, and Los Angeles where educational, cultural, social and political practices are very different. In all three countries children showed spontaneous musicality, but in the nations of Russia and the US, where formal training in music was given greater value than it was in Norway, he found reduced spontaneous music making. He insists, “It is critically important for children to master spontaneous singing, for it is part of the common code of child culture that gives them a special key to expression and human growth” (Bjørkvold, 1992, p. 63). A comparable inhibitory effect of conventions of schooling has been recorded on the spontaneous expression of religious feelings and spirituality in the early years (Hay and Nye, 1998). These innate sources of human imagining in collaborative, moral ways give value and meaning to the later cultivation of advanced cultural ideas and skills (Valiente et al., 2012).

The importance of valuing both the child’s innate musical creativity and introducing a child into his musical culture so that he may thrive within it and contribute to it can be conceptualized as a balance between two educational necessities – providing a social environment where a child’s own skills and abilities are nurtured, and a place where training is provided into the ways of a particular culture (Rogoff, 2003). Both build enthusiasm for cultural participation. This balance has been presented by Bowman (2012) and others as a consideration of two Latin roots for the English word ‘education.’ One, *educare*, means to train or to mold. Its motivation is the initiation of a person into cultural conventions, without which a person is left unable to live effectively within a particular culture, using its tools to communicate. The other, *educere*, means to lead out, or draw out. Without this more responsive nurturing, the person is left unable to engage with situations and solve problems not yet imagined. Their ability for creativity is compromised. These very different concepts of what education means are often experienced in schooling as being in tension, with *educare* often winning out, leaving the child with dry knowledge rather than living abilities supported by their own innate skills.

We propose that teachers and students of music at all levels learn how best to do their work by deliberately invoking the rhythms of the student’s innate creative vitality while demonstrating cultural conventions that make rich use of this talent (Flohr and Trevarthen, 2008). Infants and toddlers make imaginative musical play in affectionate friendships with parents or peers (Custodero, 2009); primary school children build relationships with the invention of stories in groups with free instrumental play and dance (Fröhlich, 2009); and an advanced music student is assisted to master their instrument through their teacher encouraging their playing to be like a dance representing a narrative, rich in expressive feelings (Rodrigues et al., 2009). In all instances the motives of the learner, and how they may change with development of the body and experiences gained, are of crucial importance (Bannan and Woodward, 2009; Ingold, 2018). As with all education, the success of teaching depends on recognition of how children’s ‘zest for learning’ (Whitehead, 1929; Dewey, 1938) changes with age and the development of body and mind.

We end this section with a quote from Bowman on the broader role of music *in* education. In times where the arts are often considered of marginal importance in education, it talks to the richness of our engagement with music in nurturing all learning experiences:

“The distinctive educational and developmental potential of music lies, I submit, in dynamic, bodily, and social natures, and distinctly ethical, responsive, and responsible kinds of know-how these afford. Practical knowledge is action embedded knowledge, quite distinct from theoretical knowledge and technical know-how. It is a kind of character-based sense of how best to proceed in situations where best courses of action cannot be determined by previous ones. This ability to discern the right course of action in novel, dynamic situations is precisely the kind of human asset required in today’s rapidly changing world. And musical engagements may, under the right circumstances, nurture this capacity in ways unmatched by any other human endeavour.” (Bowman, 2012, p. 31).

## Communicative Musicality and Resilience of the Human Spirit

As Daniel Stern has written (Stern, 2010), the human body has a rich range of gestural ‘forms of vitality’ – we move in musical ways. And within each actor there is both ‘self-sensing’ and ‘other-sensing’ of the degree of grace, or biological efficiency (Bernstein, 1967) and hopefulness (Trevarthen and Malloch, 2017a) in the gestural narratives of our projects. These qualities of vitality, or well-being, transmitted to others, become the qualities of relationships and social activities – their moral values (Kirschner and Tomasello, 2010; Narvaez, 2014; Trainor and Cirelli, 2015). They convey relational feelings for the degree of consensuality or sharing of expression in moving. Effort to manage the grace and morality of movements can be cultivated to assist well-being of those whose actions are confused or fearful - that is, the making or ‘poetry’ (from the Greek *poiein*, to make) of their movements may be enhanced to provide responsive and relational care or therapy (Hobson, 1985, ch3; Stern, 2000, p. xiv; Osborne, 2009b; Meares, 2016).

At times our healthy ability for graceful gesturing is met with circumstances that do not allow it to be expressed with its natural healthy vitality. For example, failure to gain a sympathetic appreciation of their musicality can cause an infant to express withdrawal and distress (Murray and Trevarthen, 1985). Instead of joyful pride in sharing play they show sadness and shame (Trevarthen, 2005b). However, an infant’s communicative musicality can also be expressive of resilience and determination.

**FIGURE 5:**
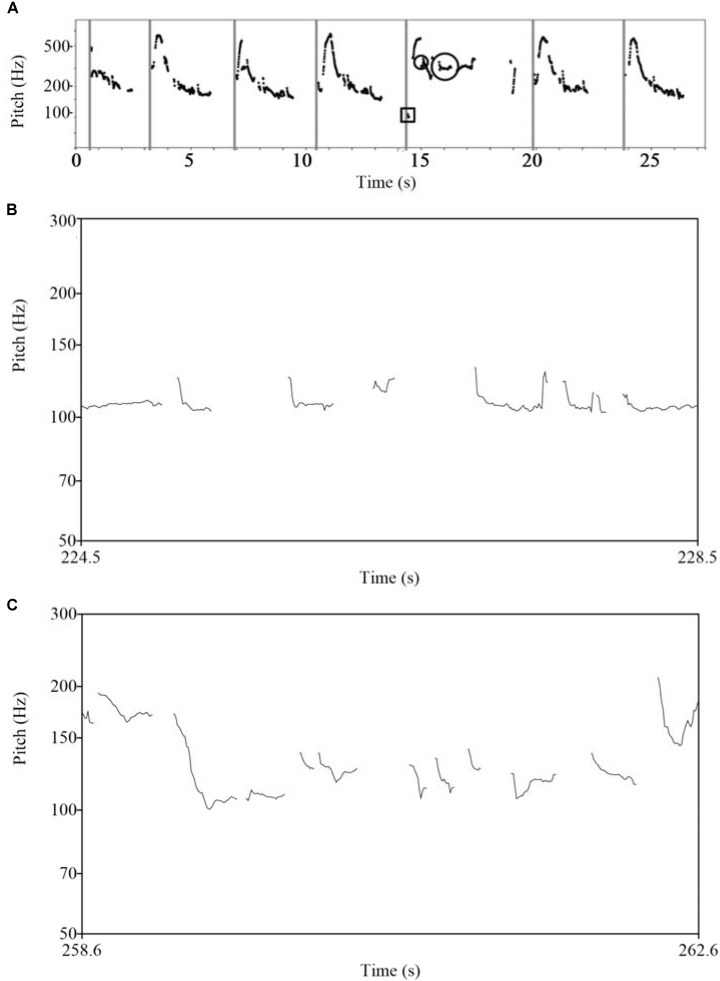
Voice modulations that express emotions of relating in a psychological disorder, and in relational therapy. **(A)** A pitch plot of a mother suffering from BPD (borderline personality disorder) speaking to her infant. She repeats the same phrase with monotonous intonation, making a slight reaction to critically timed sounds made by the infant, but cannot establish shared affective engagement. **(B,C)** Changes in the prosody of a client’s speaking in communication with a therapist, before and after a therapeutic change, with improvement in self-confidence. **(A)** Original in Gratier and Apter-Danon (2009), Figure 14.5, page 319. Reproduced with permission. **(B,C)** Originals in Malloch (2017), Figures 4.1 and 4.2, p.72. Reproduced with permission.

In the example presented in **Figure 5A** we see a consistent rigidity of expression and a lack of self-confident invention on the part of a mother suffering from BPD (borderline personality disorder). She repeats the same up-and-down vocal gesture again and again, with almost no vocal participation on the part of the infant. Where the infant does participate (shown by vocalizations with either a square or circle around them), the infant appears to be setting up the possibility for a dialog – vocalizing exactly on the ‘bar-line’ (bar 5, shown by a square) and then around the mother’s pitch (shown by a circle). Indeed, the infant’s vocalizations persuade the mother out of her repetitiveness – the mother momentarily takes notice of her infant and responds to her infant’s conversational offering by ceasing her unresponsive repetition and vocalizing once more at the infant’s pitch. But the dialog almost immediately breaks down, and the mother returns to her stereotypical, repetitive vocal gesturing.

As well as showing inflexible, ‘non-graceful’ behavior of a mother who is suffering from BPD, this example also shows the resilience and hope of the infant in the face of her mother’s rigid communicative style. This will of our communicative musicality, evident from the earliest of interactions with infants (Papoušek, 1996), is utilized in therapies that employ in-the-moment intersubjective interactions as means for healing (Stern et al., 1998; Stern, 2000, p. xiv). This immediately responsive interaction may be through talking, through music-making, through dance, or touch. The common element is an individual who is highly skilled in attuning to nuances of interpersonal timing and gesture, and who aims to lead back to health another whose personal mindedness of time-in-the-body has become compromised through hardship, suffering, or biological disruption, perhaps leading to a sense of isolation and misunderstanding (Trevarthen and Malloch, 2000). The therapist joins with the person who needs help, leading them back to health and wellbeing through their own therapeutic sense of the ‘minute particulars’ in that moment of meeting (Hobson, 1985, part 2).

Olga Maratos, a developmental psychologist who pioneered recognition of the ability of young infants to imitate expressions of an attentive and supportive adult (Maratos, 1982) took part in the establishment in Athens of a residential school for children with autism, called *Perivolaki*, meaning ‘a little garden.’ It is a day-care center with facilities in beautiful surroundings that invite playful out-door activities, games with toys and creative shared occupations such as listening to and making music. They use the psychoanalytic concept of ‘transference’ of feelings or ‘unconscious desires’ to encourage sensitive intimate and consistent relations, each child having a trusting relationship with a particular member of the staff. Stability of activities is maintained, with close engagement with the parents, who are seen weekly during the first two years of the child’s stay at *Perivolaki* and every fortnight thereafter. The average length of stay is 4–5 years (Maratos, 1998).

Olga explains that staff are trained to observe the children, think about them and discuss their behavior at staff meetings “with a view to understanding whether their autistic behaviour is defensive, refusing interaction and relations because they don’t make sense for them or because they are painful, or whether there is a pervasive lack of motivation for relating and communicating. We find both conditions present, at different times, in all our children” (Maratos, 1998, p. 206). This approach, adapting a psychoanalytic treatment which avoids diagnosis of the cause of the disorder, leads to a form of active and intimate ‘relational therapy,’ which does not rely on verbal formulation of anxieties and lack of trust.

Affect attunement has been defined as qualities of vocal and body gesture that carry meaning in parent–infant communication – it is, “the performance of behaviours that express the quality of feeling of a shared affect state, but without imitating the exact behavioural expression of the inner state” (Stern, 1985, p. 142). This largely unconscious ‘recasting’ of events is necessary to “shift the focus of attention to what is behind the behaviour, to the quality of feeling that is being shared” (Stern, 1985, p. 142). We say the relationship is now one of ‘companionship,’ a word from Latin meaning ‘to break bread with’ and defined here as the wish to be with an other for a mutually beneficial ‘inner’ purpose, apart from reasons of immediate survival, procreation or material gain. Companionship involves exchanging affect through sharing the quality or virtue of impulses of motivation, which is the original rich meaning of ‘sympatheia’ in Greek (Trevarthen, 2001).

The therapeutic relationship, even in talking therapies focussed on what the language says about feelings, is underpinned by the manner or graciousness of our gestural exchanges – whether those qualities are carried in vocal prosody or bodily movements (Stern, 2000, p. xiv; Malloch, 2017). It is about the direct and desired sharing of feelings in human vitality. Stern (2010) writes eloquently on the importance of listening for the forms of vitality (the forms of feeling as Hobson, 1985, called them) that are being expressed through prosody – “without the dynamic vitality features of the intention-unfolding process we would not experience a vital human being behind the words that are being said” (Stern, 2010, p.124). He also writes of the use of metaphors as carriers of images of our in-the-moment vitality (also see Hobson, 1985, ch.4). For example, in the work of Stephen, the first author, as a therapist, here is the text of an exchange between him and a client discussing the client’s sense that his life has never measured up to his notion of what a successful life should look like:

Client: My father thought I was stupid. He’d call me ‘stupid boy.’ Therapist: The way you said that – perhaps a sort of exhaustion and emptiness – reminds me of the tide going out in a bay. Client: Empty… like something’s slowly leaving.

Here the focus on the vitality that the client associates with his father’s dismissing statement leads to a new experience to explore therapeutically – something is slowly leaving. Further discussion focussed on the experience of what ‘slowly leaving’ feels like. When the therapist works like this, “we move from an enquiry about intentions, means, and goal states to an enquiry about processes of creation, emerging, and becoming” (Stern, 2010, p.126). We move from distanced recollection or speculation to life in the present moment (Stern, 2004).

The prosody of the client’s voice sometimes sums-up the therapeutic change itself. In the example below of Stephen’s work a client is talking of an emerging ‘new me’ in contrast to an ‘old me.’^2^ The ‘old me’ was marked with ‘a lack of self-respect.’ ‘I blame myself when things go wrong, I believe I’m not working hard enough.’ The voice is drone-like, body hardly moving. **Figure 5B** shows a four-second section of a pitch plot of ‘old me’ voice.

After describing ‘old me’ the client’s body relaxed, they looked up from the floor, hands lifted from their lap, the volume of their voice increased, its pitch lifted, and they began talking of ‘new me.’ ‘New me is more rational about life. This part says, “Well, I was uncommunicative this morning – that’s all right, that’s OK. That’s just the way I was. Doesn’t make me a bad person. Other times I communicate really well!” **Figure 5C** is a four-second pitch plot of ‘new me.’ The shift in the vitality of the musicality is clear. Stephen *felt* the distinct difference in the vitality of the two “me’s” of the client, and continued the session exploring how the new “me” might express itself in the world (see Malloch, 2017, for further discussion of communicative musicality in therapy).

The role of our communicative musicality in supporting our wellbeing lies at the very heart of the practice of using music therapeutically. Music therapy covers a wide range of ways of using musical experiences – stories in sound – to heal and improve people’s lives. The Australian Music Therapy Association defines Music Therapy as: “a research-based practice and profession in which music is used to actively support people as they strive to improve their health, functioning and wellbeing.” It is the compassionate use of music to engage another emotionally, interpersonally, cognitively, and culturally. “Music is therapeutic because it attunes to the essential efforts that the mind makes to regulate the body, both in its inner neurochemical, hormonal and metabolic processes, and in its purposeful engagements with the objects of the world, and with other people” (Trevarthen and Malloch, 2000). This is particularly so during improvisational music therapy, where the therapist supports the client towards change – greater integration of experience and freedom in communication (Pavlicevic, 2000, ch.6; also see Malloch et al., 2012, on the effectiveness of improvisational music therapy with neonates).

However, the practice of music therapy is more than the therapeutic use of preverbal protomusic, however, important this is. Reflecting our discussion above on music and education, music therapy is also making use of the cultural forms of our musicality, and the power these cultural forms have within our psyche (for example, Donald, 1991; MacDonald et al., 2002; Stern, 2010).

A relationship between our communicative musicality and our culturally made music for the practice of music therapy is proposed by Pavlicevic and Ansdell (2009). They emphasize the peculiarly *musical* relationship established within music therapy practice – that is, that the cultural elaboration of communicative musicality relates to our communal, social lives. Music therapy engages our shared communicative musicality, and welcomes us into the shared cultural, communal experience of musicking, using the tools of a particular cultural type of music – one of many musics in the world (Stige, 2002).

Thus, part of the reason music therapy ‘works’ is its invitation for cultural collaboration – we exercise what has been called our ‘deep social mind’ (Whiten, 2000) following particular cultural forms. This wish to learn the forms of culture, our ‘conformal motive’ (Merker, 2009), comes to life within an environment where we sense our communicative gestures are being valued by another or others through the creation of shared narratives of vitality forms (Stern, 2010). We feel ourselves to be both a companion in our shared narrative of communicative embodied gestures and a companion in a particular shared cultural collaboration. It is within this dual companionship that our deep yearning to belong is met and satisfied, and where healing can occur.

## Musical Affections of the Embodied Human Brain

In our opinion brain science has been most insightful into the nature of the self and what makes us human, and how we share the joy and pains of life, when it investigates ‘Primary Process’ emotional guidance of brain growth for regulation of vitality in body movement and its ‘seeking’ awareness (Hess, 1954; Solms and Panksepp, 2012). It offers us insight when it investigates how experiences develop by generating expectations of well-being in companionship and by enriching it with cultural meaning (Trevarthen, 1990b). It shows that differences in the rate of development of cognitive processes in left and right hemisphere at different ages are caused by different affections (Thatcher et al., 1987; Chiron et al., 1997), from which arise correlations with musical behaviors and other creative forms of play, as well as Piagetian stages of rational mastery of the body and objects it uses, and development of language.

The neuroscientist Jaak Panksepp, who studied the emotions of mammals who do not cultivate music, but who use patterns of movement including signs with sound to regulate their social lives in ways that anticipate our richer experience of the sounds of our movement using the tools of culture, offers insight into why we are musical beings (Panksepp and Bernatzky, 2002). And in a recent synopsis he agrees that mastery of language in early years depends on the sense of purpose we share in musical ways:

“Human languages are coaxed into the brain, initially by the melodic intonations of motherese by which emotional communication becomes the vehicle for propositional thought.” (Panksepp et al., 2012, p. 11).

Like the brain of any animal, the human brain grows to represent and regulate a body form in movement (Trevarthen, 1980, 2001, 2004). And from even before birth, the self-formation of a personal self in the brain of the fetus is led by manifestations of movement.

“The first generalized movements occur in week 8 (de Vries et al., 1984), but already in week 5 monoamine transmission pathways grow from the brainstem to animate the primordial cerebral hemispheres. Key components of the Emotional Motor System (hypothalamus, basal ganglia and amygdala) are in place when the neocortex is unformed.” (Trevarthen, 2001, p. 26).

After the baby is born and seeks intimate communication of all motives with a parent, the affective system remains as the director of learning and appreciation of what is gained by new awareness.

Psychiatrist and literary scholar Iain McGilchrist in *The Master and His Emissary* (McGilchrist, 2009) has presented a brilliant review of behavioral and brain research, and a clear conception of complementary consciousness in the two cerebral hemispheres. “Music”, he writes, “being grounded in the body, communicative of emotion, implicit, is a natural expression of the nature of the right hemisphere” (p. 72).

McGilchrist’s research leads him to the political view that we are living in a society that grants too much power to the special refined perceptuo-motor and scientific skills of the left brain, while failing to appreciate how the right brain gives purpose and value to all that we do, thus pointing to the importance of closer cooperation between science and the fundamental values of the humanities. He draws on anthropological information about the universal principles of social understanding at very different levels of technical ability and manufacture, and on the importance in all social groups and cultures of musical performances, which he concludes, from a wide range of evidence, that music evolved before language and contributed to the formulation of its syntax and prosody (see Sachs, 1943).

Music, he says, was not,

“an irrelevant spin-off from something with more of a competitive cutting edge – namely, language…. rather the reverse. If language evolved later, it looks like it evolved from music…. Rousseau in the eighteenth century, von Humboldt in the nineteenth century and Jespersen in the twentieth, have thought it likely that language developed from music…. That we could use non-verbal means, such as music, to communicate is, in any case, hardly surprising. The shock comes partly from the way we in the West view music: we have lost the sense of the central position that music once occupied in communal life, and still does in most parts of the world today…. We might think of music as an individualistic, even solitary experience, but that is rare in the history of the world.” (p. 104).

And he quotes neurologist Oliver Sacks, who said:

“This primal role of music is to some extent lost today, when we have a special class of composers and performers, and the rest of us are often reduced to passive listening. One has to go to a concert, or a church or a musical festival, to recapture the collective excitement and bonding of music. In such a situation, there seems to be an actual binding of nervous systems, the unification of an audience by a veritable ‘neurogamy’ (to use a word favoured by early Mesmerists) (Sacks, 2006, p. 2528).

Twenty-six years before McGilchrist published his book, the anthropologist Victor Turner, famous for his book *From Ritual to Theatre*, drew on knowledge of the different functions of the hemispheres to identify play with a collaboration between them, in an article entitled “Play and drama: The horns of a dilemma”:

“Current ideas about differences between the left and right hemispheres of the brain provide a basis for speculating about the nature of play. Play encompasses both the rationality and order of the left hemispheric orientation, and the improvisation and creativity of the right. But play also transcends these oppositions, running rings about them as it encircles the brain’s consciousness” (Turner, 1983, p. 217).

Good, beautiful and enjoyable, music is created out of poetic play. Indeed, we *play* music (Trevarthen and Malloch, 2017b).

A sense of time in the mind is the fabric from which movements of all kinds are woven into ambitious projects that value elegance with efficiency. It is a manifestation of the ‘biochronology’ that is essential to the vitality of all forms of life (Osborne, 2017). In *Rhythms of the Brain*, György Buzsáki (2006) presents a wealth of evidence that the brain functions as a coherent rhythmic system, always in synch., and with a rich array of rhythms that are organized to collaborate.

“At the physiological level, oscillators do a great service for the brain: they coordinate or ‘synchronize’ various operations within and across neuronal networks. *Syn* (meaning same) and *chronos* (meaning time) together make sure that everyone is up to the job and no one is left behind, the way the conductor creates temporal order among the large number of instruments in an orchestra” (Buzsáki, 2006, p. viii).

From Panksepp and Trevarthen (2009), p. 114:

“Music is performed with the measure of expressive movements in time, and with tensions created by combining rhythms (Osborne, 2009a). The ‘architecture’ and ‘narration’ of moving in psychological time is also displayed with emotional qualities related to vital functions of the body (Trevarthen, 2009). These psychobiological elements of vitality are charted in three bands or ranges of physical or scientific time: (1) for the felt and imagined ‘extended present’ (from 10 seconds to years); (2) for the conscious ‘psychological present’ (Stern, 2004), with its serially ordered steps of motor control coupled to the physiological rhythms of breathing and variations in heart rate (0.3 to 7 seconds); and (3) for ‘reflex experiences’ and ‘just noticeable differences’ too fast to be regulated by movements that are prospectively controlled in awareness (5 to 200 milliseconds). (For detail and the sources of this description see Trevarthen, 1999).

The time of musical narrative, which Imberty (2000) calls the macrostructure or ‘story-without-words’ of music, is related to the times of expressive behavior that form ‘protonarrative envelopes’ of intuitive vocal and gestural play between infants and their mothers (Stern, 1985, 1995; Malloch, 1999). The period corresponding to a stanza or verse of 20 to 40 seconds may be manifested in the brain, as gamma waves or parasympathetic cycles, which control autonomic functions of the heart and breathing. It continues to be active through sleep to produce fluctuating rates of breathing and heartbeat, as well as electrical activity of the cerebral cortex that might be related to the rehearsal and consolidation of memories in dreaming (Delamont et al., 1999). In wakefulness the narrative cycle is charged and modulated for intersubjective meaning with the ‘microtonal’ and ‘microtemporal’ variations of emotion that express urgency and facility in skilled control of moving within the voice of a singer or the playing fingers of an instrumental performer, and in the hearing of a listener (Imberty, 1981, 2000; Gabrielsson and Juslin, 1996; Juslin, 1997, 2001; Kühl, 2007; Osborne, 2009a). Music can assist the synchronization of physiological functions of respiration and heart activity and bring improvement in locomotor activity, and it can improve cognitive and memory processes by brain synchronization.”

Rhythmic co-ordination by the Intrinsic Motive Pulse (IMP) of the brain holds body movements together in composition of intentions and experiences (Trevarthen, 1999, 2016). It is the medium for all shared experiences and purposes, and for the convivial vitality of music making.

## CODA: The Philosophy of Human Vitality

In her review of the role of movement and sense of time in the creation of intelligence, Barbara Goodrich, as a philosopher, traces a history of ideas supporting the view that consciousness is founded on emotions for agency, which we argue are the *sine qua non* for music. In opposition to the “implicit philosophical presuppositions inherited from the canon of Plato, Aristotle, Descartes, and Kant, e.g., that consciousness is self-reflective, passive, and timeless,” she proposes a natural science view.

“Western philosophy, however, also includes what might be described as a counter-tradition—and one that is more compatible with empirical biological science than the usual canon. Heraclitus, Spinoza, Schopenhauer, Nietzsche, and especially the 20th century French philosopher and psychologist, Merleau-Ponty, all anticipated aspects of Llinás’s and Buszáki’s approaches… sketching out a notion of consciousness emerging from motility, and generating new hypotheses for neurophysiological research.” (Goodrich, 2010, p. 331).

We have argued that music comes from this very foundation of consciousness in motivated motility, and we underline the importance of a philosophy that acknowledges the motives and feelings of our life, as well as the intelligence we show in relating to persons, other life forms, and objects in our environment, by recalling the achievements of the philosophers of the Scottish Enlightenment – Hutcheson, Hume, Smith and Reid. In line with Goodrich’s “counter tradition,” their work anticipates the new understanding of the human BrainMind pioneered by Panksepp and Damasio, which gives primary importance to feelings of vitality in movement, and to emotions that express positive and negative affections in sympathetic communication. This is the science of communicative musicality which underpins the music we create and enjoy.

The Scottish philosophers of the 18th Century, led by Francis Hutcheson, held that relationships and social life depend upon a universal human capacity for “innate sympathy,” which generates a conscience, a sense of beauty, a public ‘common sense’ that values happiness and is disturbed by misery, and a moral sense that perceives virtue or vice in ourselves or others (Hutcheson, 1729, 1755; Hume, 1739–1740).

Smith (1759) in his *Theory of Moral Sentiments* took ‘sympathy’ to designate any kind of ‘moving and feeling with,’ whether motivated positively or negatively, and including posturing and acting in the same expressive way as another’s body (cf. the work of Stern on ‘affect attunement’ quoted earlier), and he also imagined experiences of relating and being sensed, as, for example, interrogating one’s conscience.

He said:

“How selfish soever man may be supposed, there are evidently some principles in his nature, which interest him in the fortune of others, and render their happiness necessary to him, though he derives nothing from it except the pleasure of seeing it.”

“Sympathy… may…, without much impropriety, be made use of to denote our fellow-feeling with any passion whatever.” *Part I – Of the Propriety of Action; Section I – Of the Sense of Propriety, Chapter I – Of Sympathy.*

He examined his conscience to understand being a person in relations.

“When I endeavour to examine my own conduct, when I endeavour to pass sentence upon it, and either to approve or condemn it, it is evident that, in all such cases, I divide myself, as it were, into two persons; and that I, the examiner and judge, represent a different character from that other I, the person whose conduct is examined into and judged of. The first is the spectator, whose sentiments with regard to my own conduct I endeavour to enter into, by placing myself in his situation, and by considering how it could appear to me, when seen from that particular point of view’. The second is the agent, the person whom I properly call myself, and of whose conduct, under the character of a spectator, I was endeavouring to form some opinion.”

This picture of a duplex mind regulated by motives of sympathy anticipates the distinction made by William James in 1892 and by Martin Buber in 1923 between a fundamental “I-Thou” state of awareness and the objective “I-It” relations with the physical word we acquire in communication.

Otteson, joint professor of philosophy and economics, and chair of the Philosophy Department, at Yeshiva University, and adjunct Professor of Economics at New York University, has proposed that Adam Smith’s *Theory of Moral Sentiments* (1759) has a more profound message for commerce and industry than *The Wealth of Nations*.

“Smith’s picture thus has a clear anti-Freudian thrust: it denies the hydraulic picture of human emotions according to which emotions build up “pressure” that must be “released.” Instead, and more plausibly, it conceives of emotions as things that can be controlled and trained by exercising what Smith calls “self-command.” The activity of reciprocal adjustment is then repeated numberless times in every person’s lifetime, as it is between and among the people in one’s community, resulting in the creation of an unintended and largely unconscious system of standards. These standards then become the rules by which we determine in any given case what kind of behavior is, as Smith calls it, “proper” in a situation and what “improper”—meaning what others can reasonably be expected to enter into.” Otteson (2000, November 01).

Smith was a great admirer of the messages of music and wrote about the communication of its poetic massages in his essay *Of the Nature of that Imitation which takes place in what are called the Imitative Arts*, published in 1777 (Smith, 1777/1982).

Beginning with his *A Treatise of Human Nature* (1739), David Hume strove to create a natural science of human psychology in opposition to René Descartes’ rationalism. He concluded that desire rather than reason motivates our behavior. Anticipating Merleau-Ponty’s phenomenology he also argued against the existence of innate ideas, concluding that we know only what we directly experience. He held that inductive reasoning and causality cannot be justified rationally, rather we follow custom and constant relations between ideas rather than logic. He concluded that we do not have a ‘conception of the self,’ only sensations of being alive. Following his teacher Hutcheson (1729), he believed that ethics are based on feelings rather than abstract moral principles.

Finally, there is a bold clarity in the work of Thomas Reid, the third great follower of the teachings of Hutcheson, and a vigorous debating companion to David Hume. He wrote *An Inquiry into the Human Mind on the Principles of Common Sense* (Reid, 1764).

Reid founded the Scottish School of Common Sense. For him ‘common sense’ is based on a direct experience of external reality, experience that becomes internal in language, which is based on an innate capacity pre-dating human consciousness, and acting as an instrument for that consciousness. He distinguished the acoustic element from the meanings which seem to have nothing to do with the sounds as such, a state of language, which he calls ‘artificial,’ that cannot be the primeval one, which he terms ‘natural.’ He described the way a child learns language by imitating sounds, becoming aware of them long before he or she understands the meaning in the artificial state of contemporary adult speech. If, says Reid, children were to understand immediately the conceptual content of the words they hear, they would never learn to speak at all. Here Reid distinguishes between natural and artificial signs.

‘It is by natural signs chiefly that we give force and energy to language; and the less language has of them, it is the less expressive and persuasive…. Artificial signs signify, but they do not express; they speak to the intellect, as algebraic characters may do, but the passions and the affections and the will hear them not: these continue dormant and inactive, till we speak to them in *the language of nature*, to which they are all attention and obedience.’ (Reid, 1764, p. 52).

‘Language of nature’ we equate with our embodied moving consciousness – our communicative musicality. An excess of ‘artificial signs,’ perhaps aimed at increasing productivity, leads to loneliness and ruthless rationality. However, the cultivation of our communicative musicality, in ourselves and others, through playful music, dance, ritual and sympathetic companionship, makes our communal life of shared work of the body and mind creative in more hopeful ways. It restores our common humanity and our connection with all living things.

## Ethics Statement

Informed consent was gained for all data presented in this paper.

## Author Contributions

SM contributed to all sections of the paper, particularly sections Communicative Musicality and Education into the Culture of Music and Communicative Musicality and Resilience of the Human Spirit. CT contributed to all sections of the paper, particularly sections The Genesis of Music in Infancy – A Short History of Discoveries, Case Studies of Infant Musicality, and Musical Affections of the Embodied Human Brain.

## Conflict of Interest Statement

The authors declare that the research was conducted in the absence of any commercial or financial relationships that could be construed as a potential conflict of interest.

## Footnotes

1 We regard the term ‘infant directed,’ usually used in this context, to be a misnomer that does not acknowledge the co-operative and shared nature of the interaction. We use ‘infant-inclusive’ to refer to the style of speech and singing that spontaneously occur between caregiver and infant.

2 Written consent was obtained from the client by the first author.
